# Fast algorithms for singular value decomposition and the inverse of nearly low-rank matrices

**DOI:** 10.1093/nsr/nwad083

**Published:** 2023-03-25

**Authors:** Chen Xu, Weiwei Xu, Kaili Jing

**Affiliations:** Department of Mathematics and Statistics, University of Ottawa, Canada; School of Mathematics and Statistics, Xi’an JiaoTong University, China; School of Mathematics and Statistics, Nanjing University of Information Science and Technology, China; Department of Mathematics and Statistics, University of Ottawa, Canada

## Abstract

This perspective focuses on the fast algorithm design for singular value decomposition and inverse computation of nearly low-rank matrices that are potentially of big sizes.

## PROBLEM

Matrix computation is a fundamental task in science and engineering; it is recognized as one of the seven computational ‘giants’ of massive data analysis [1], analogous to the seven ‘dwarfs’ in high-performance computing. In recent years, as the scale of datasets rapidly grows, analysts and engineers frequently need to deal with big matrices with a nearly low-rank structure. The big size of a matrix brings great numerical hurdles to matrix computation. In this work, we focus on two typical tasks in matrix computation: singular value decomposition (SVD) and matrix inverse on nearly low-rank matrices that are potentially of big sizes.

For the convenience of discussion, we denote by **C**^*m*×*n*^ the family of complex matrices with *m* rows and *n* columns. For any *A* ∈ **C**^*m*×*n*^, let *r* be its rank and σ_*i*_, *i* = 1, 2, …, *k*, be the set of its singular values with *k* = min{*m, n*} and σ_*i*_ ≥ σ_*i*+1_. Matrix *A* is said to be of low rank if *r* < *k* and nearly low rank if


\begin{eqnarray*} \frac{\sum _{i=1}^{r}\sigma _{i}^{2}}{\sum _{i=1}^{k}\sigma _{i}^{2}} \ge 1-\epsilon \end{eqnarray*}

for some small threshold ε > 0.

When *A* is of nearly low rank, only *r*-out-of-*k* singular values are significant; it can be thus approximated by an *r*-rank matrix $\tilde{A}$ with all insignificant singular values disregarded. It is known that finding $\tilde{A}$ amounts to solving an *r*-truncated SVD of *A* with a user-specified value of *r*. When *m* = *n*, the techniques used in finding $\tilde{A}$ may also be applied to solve the inverse of *I* + *A* with *I* being an *m* × *m* identity matrix. This was Task 5 in the Arena Contest of the 2022 International Algorithm Case Competition (http://112.74.40.78:8084/).

Solving the SVD or inverse of a nearly low-rank matrix has a wide scope of applications in many scientific areas. For example, in high-dimensional statistics, the number of covariates is larger than the sample size; this naturally leads to a low-rank sample covariance matrix being handled in tasks like regression or principal component analysis. In massive data analysis, the Gram matrix of a kernel method often comes with a nearly low-rank structure due to a large number of correlated entries. In the field of communication, the transmission speed of a multi-antenna wireless system largely relies on the efficiency of solving a series of SVD and inverse problems on the channel information matrices. Since the communication channels are highly similar among the users, the corresponding channel information matrices are typically of nearly low rank.

Solving the SVD or inverse can be numerically costly for big matrices; the task comes with a typical complexity of *O*(*mn*^2^) or *O*(*m*^3^), respectively. For an *r*-rank matrix, techniques such as QR decomposition can be applied to transform the original *m* × *n* problem into *r* × *r* problems [2]. These techniques are helpful in reducing the complexity of the task only when the rank *r* is known beforehand; this, however, is unrealistic in many applications, where *r* is typically a ‘to-be-determined’ value. Moreover, the existing methods to solve the SVD or matrix inverse often suffer from algorithmic instability due to multiple-root issues. All these call for new techniques to handle this functional task, to which we now turn.

## ALGORITHMS

We tackle the considered problem by a novel procedure. Our ideas are to apply a new on-line low-rank decomposition tool, called QB decomposition, to transform the original *m* × *n* high-order problems into *r* × *r* lower-order problems, and develop an random renormalization theory to solve the rank *r* unknown problem. The three strategies adopted are as follows.

### Strategy 1: QB decomposition

The low-rank decomposition is a basic tool of complexity reduction of matrix computation, which aims to decompose a matrix *A* ∈ **C**^*m*×*n*^ into the form *A* = *QB*, where *Q* ∈ **C**^*m*×*r*^ is a column orthogonal matrix and *B* ∈ **C**^*r*×*n*^ a structured matrix (normally, an upper triangular matrix). The classical tools used are QR decomposition and the Gram-Schmit process (GSP). The QR decomposition is efficient for fully rank matrices and has *O*(*mn*^2^) complexity, while GSP can generate a perfect *Q* but a partial *B* because of missing the expression coefficients of columns relevant to previous columns. Based on the observation that *B* = *Q*^*H*^*A* holds for every low-rank decomposition, we skillfully arrange the computation order and propose a revised GSP procedure; see Algorithm 1. We call such low-rank decomposition the QB decomposition of matrix *A*.

**Algorithm 1:** (QB decomposition)

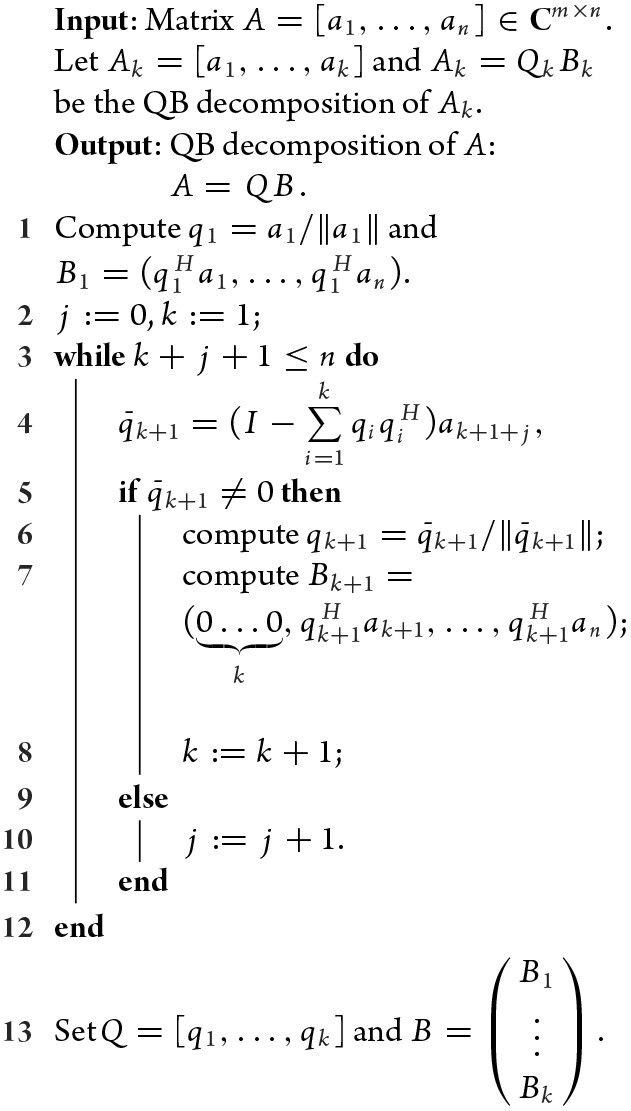

The QB decomposition is an *O*(*mnr*) complexity procedure, which can be conveniently used to transform the high-order SVD problem of *A* ∈ **C**^*m*×*n*^ into a lower-order SVD problem of *C* ∈ **C**^*r*×*r*^, where *r* is the rank of *A*. The procedure, say, can be as follows: decompose *A* = *QB, AA*^*H*^ = *QBB*^*H*^*Q*^*H*^, and then the SVD of *A* can be obtained from the SVD of *C* = *BB*^*H*^, where *C* is an *r* × *r* matrix.

The QB decomposition has a very natural ‘online correction’ property that benefits reduction of storage and data movements in implementation. In particular, when one more column, say, *a*_*k*+1_, is added into *A_k_* = *Q_k_B_k_*, the corresponding QB decomposition *A*_*k*+1_ = [*A_k_a*_*k*+1_], = *Q*_*k*+1_*B*_*k*+1_ can be computed through *Q_k_*, *B_k_* and *a*_*k*+1_ only. Thus, QB decomposition can essentially provide a low-rank representation of a sequence of input vectors. In this point of view, we can apply QB decomposition to sequence {*q*_1_, *Aq*_1_, *Aq*_2_, …, *Aq_r_*}, justifying the following theorem.

Theorem 1. **Theorem 1.** *Assume that *A* ∈ **C**^*n*×*n*^ is Hermitian, *q*_1_ is any fixed nonzero unit vector and that the QB decomposition of sequence {*q*_1_, *Aq*_1_, *Aq*_2_, …, *Aq_r_*} is given by*

\begin{eqnarray*} [q_{1},Aq_{1},Aq_{2},\ldots ,Aq_{r}]=Q[e_{1},B], \end{eqnarray*}

*where *e*_1_ = (1, 0, …, 0)^*T*^, *Q* = [*q*_1_, *q*_2_, …, *q_r_*]. Then *A* = *QBQ*^*H*^ is a Lanczos decomposition, i.e. {*q*_1_, *q*_2_, …, *q_r_*} is a Lanczos sequence and *B* is a triple diagonal matrix, respectively defined by the Lanczos process [3].*

Theorem 1 shows that the Lanczos process can be redefined by the QB decomposition.

### Strategy 2: Lanczos’ process revised

The Lanczos method computes the eigendecomposition of Hermitian matrices, which works by tridiagonalizing the matrices first (the tridiagonalization stage, namely, the Lanczos process), and then computing the deduced, much simplified tridiagonal eigendecomposition problems. It is a state of the art procedure while the instability and multiple-root issues have not be thoroughly resolved.

Theorem 1 bridges the QB decomposition and Lanczos process. Such a connection enables us to rationally revise Lanczos’ method to help mitigate those issues. For example, we can substantially improve the algorithmic stability by employing the QB decomposition in place of the Lanczos process, or by adding a QB update after every two steps of the construction of orthogonal rows *q_i_* in the Lanczos process. Furthermore, we proved the following theorem.

Theorem 2. **Theorem 2.** *Assume that *M* ∈ **C**^*n*×*n*^ is a Hermitian matrix and that {*q_i_*} is the sequence defined by the Lanczos process for *M*. If *q*_1_ satisfies*

(1) \begin{eqnarray*} Mq_{1}&&\ne 0 \textit{ and } q_{1} \textit{ is not an }\\ &&\textit{eigenvector of}\ M \end{eqnarray*}

*then* {*q_i_*} *is well defined, and q_i_* ≠ 0 *if and only if *i* ≤ *n*_0_, where n*_0_*is the degree of the minimal polynomial of M*.

Theorem 2 implies that Lanczos’ method (precisely, the Lanczos process) will never be terminated before *n*_0_ steps of update, and the yielded {*q_i_*} must locate at different eigensubspaces. Based on this, incorporated with an *m*_0_ times restart scheme, where *m*_0_ is the number of maximal-multiplicity eigenvalues of *A*, and choosing


\begin{eqnarray*} &&M_0 = A, \\ && M_j=\bigg (I-\sum _{l}^{j}Q_lQ_l^H\bigg )A,\\ &&\quad\qquad\,\,\, j=1, \ldots , m_0-1 \end{eqnarray*}

(where *Q_l_* is defined as in Algorithm 2), with the initial values satisfying (1) each time, we can then effectively resolve the multiple-root issue. We note that condition (1) is very easily satisfied in use due to the fact that all the vectors satisfying condition (1) form a zero measure set.

**Algorithm 2:** (Lanczos’ process revised)

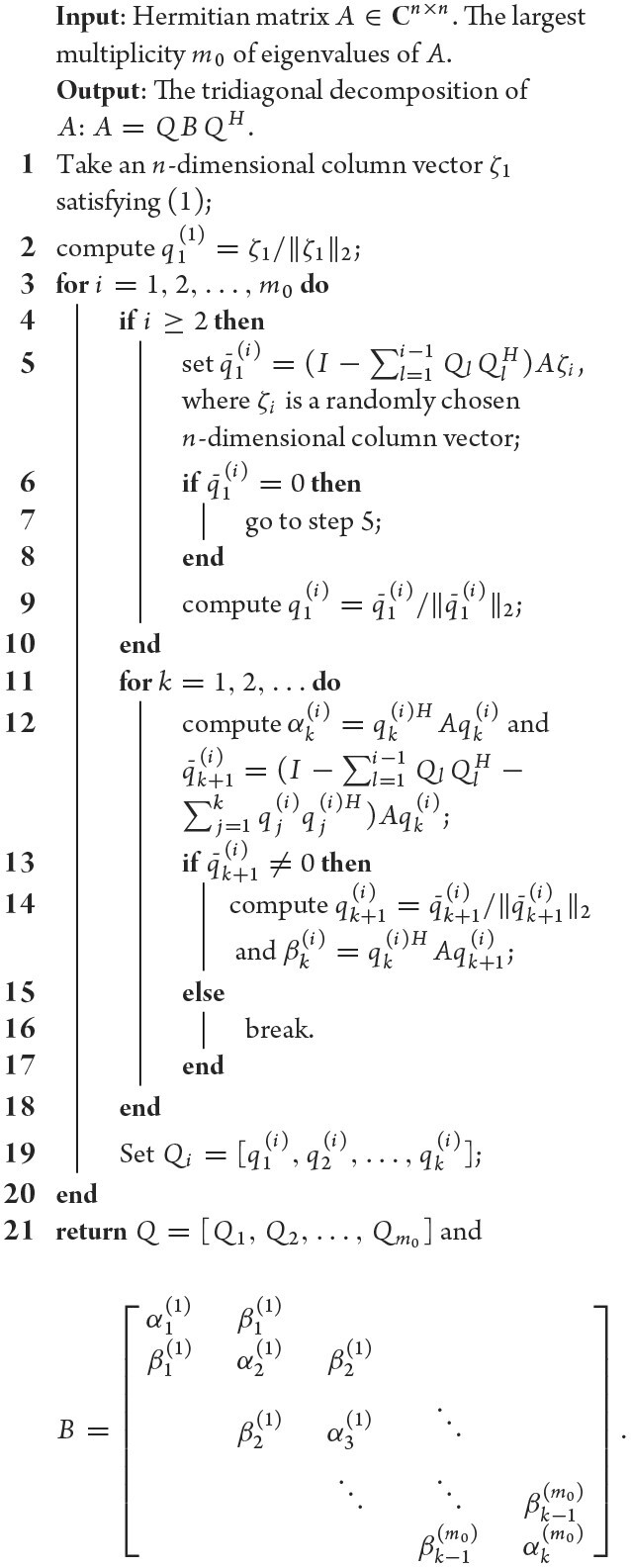

With this in wind, we propose a revised Lanczos process; see Algorithm 2. Its complexity is *O*(*n*^2^*r*).

### Strategy 3: random renormalization theory

Random renormalization is an attempt to recombine the rows of *A*, through an action of random transformation Ω (matrix), such that (i) rank(*A*Ω) = min{rank(*A*), rank(Ω)} and (ii) *A*Ω and *A* have the same low-rank basis, i.e. their QB decompositions *A*Ω = *Q*_1_*B*_1_ and *A* = *QB* have the property that *Q* = *Q*_1_. We proved that there exist random matrices, e.g. Gaussian random and Bernoulli random matrices, that satisfy the random renormalization conditions in high probability.

The random renormalization property (ii) implies that instead of *A* we can employ *A*Ω’s QB decomposition to determine the low-rank basis *Q*, and property (i) then implies that using the QB decomposition of *A*Ω, not *A*, may bring an exclusive advantage: *A*Ω keeps full column rank and the resulting upper triangular expression *B* nonsingular, i.e. its diagonal entries all are nonzero whenever rank(Ω) ≤ rank(*A*). This advantage can be utilized to resolve the rank *r* unknown problem in a more economic way. The method is as follows. We recursively define Ω_*k*+1_ = [Ω_*k*_ ξ_*k*+1_] with a random renormalization vector ξ_*k*_, *k* = 1, 2, …, i.e. Ω_*k*_ = [ξ_1_, ξ_2_, …, ξ_*k*_] and implement the QB decomposition (using the online version applied to *A*Ω_*k*+1_ = [*A*Ω_*k*_ *A*ξ_*k*+1_]), resulting in QB decompositions *A*Ω_*k*_ = *Q_k_B_k_* and *A*Ω_*k*+1_ = *Q*_*k*+1_*B*_*k*+1_. Terminate the process when an index *r* appears such that *B_r_* has no zero entries but *B*_*r*+1_ does. Obviously, the rank *r* of *A* can be identified in this way and, more exclusively, this is realized in the QB decomposition process of *A*Ω_*r*_, with no additional computation involved thanks to the online correction property of the QB decomposition. In addition, to avoid the extra computation cost of multiplication of *A*Ω in place of *A*, we suggest applying Bernoulli random renormalization in applications.

To identify the rank of *A* by counting the number of nonzero diagonal entries of *B* as above provides an opportunity to treat SVD problems of low-rank and nearly low-rank matrices in a unified way. In effect, assuming that *B*(*i, i*) is the *i*th diagonal element of *B* and that ϵ_1_ is a tolerance threshold, we can regard *B*(*i, i*) as zero if and only if |*B*(*i, i*)| ≤ ϵ_1_, where ϵ_1_ is zero when *A* is of low rank and a small threshold value when *A* is of nearly low rank. We proved that ε_1_ can be specified explicitly by the given tolerance ε.

Incorporated with the three strategies above, the finalized algorithms we suggested for fast computation of the SVD and inverse of nearly low-rank matrices are presented in Algorithms 3 and 4, whose complexities are *O*(*mnr*) and *O*(*n*^2^*r*), respectively.

**Algorithm 3:** (SVD)

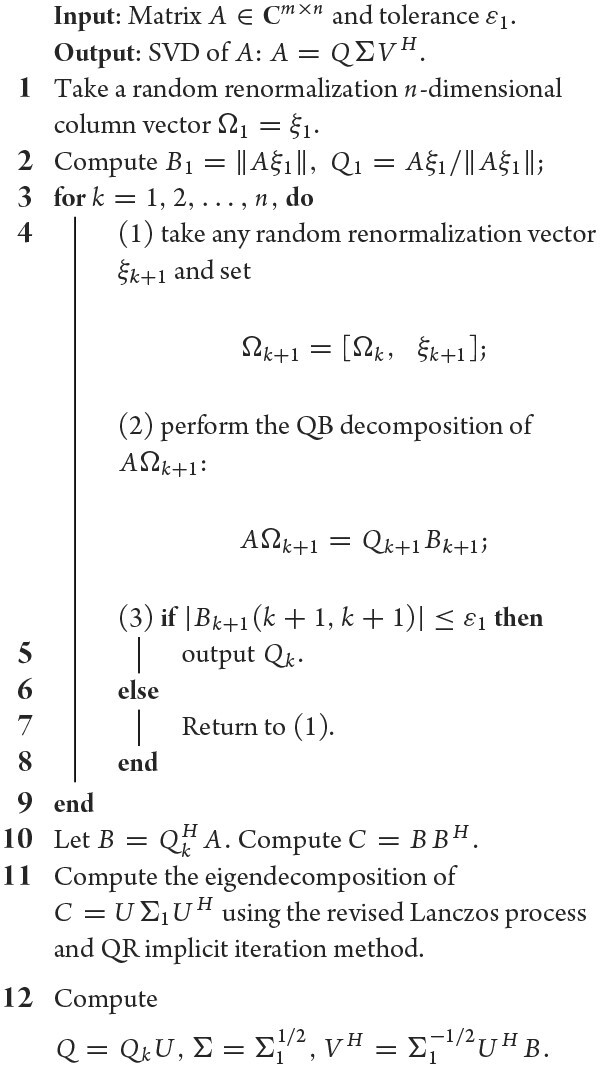

**Algorithm 4:** (Inverse of (*I* + *A*))

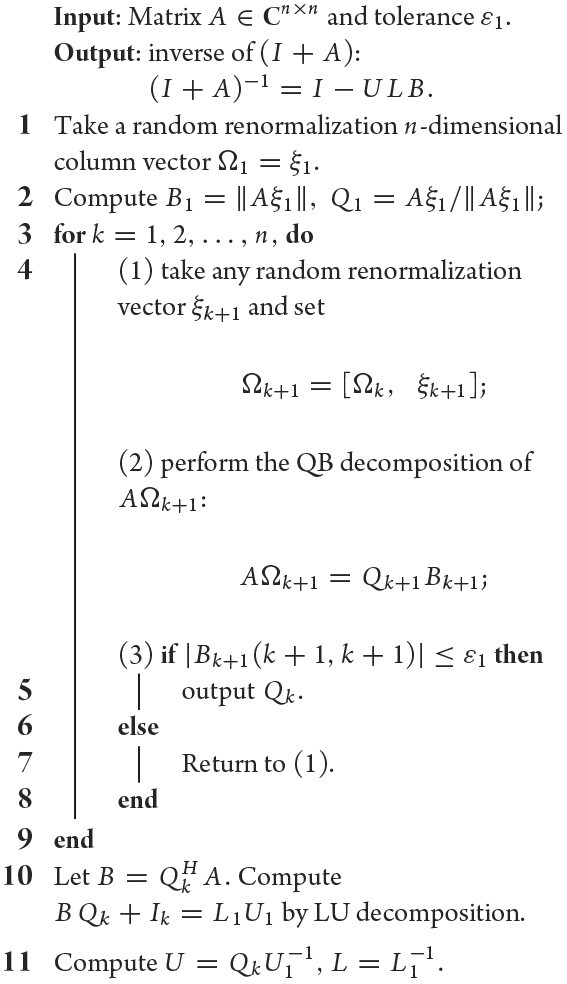

The proofs of Theorems 1 and 2 are given in the online supplementary material.

## EVALUATION

The proposed method is numerically tested with simulations conducted in Matlab^®^. In particular, we access Algorithms 3 and 4 using a randomly generated *m* × *n* matrix *A*, where *A* = *U*Σ*V*^*H*^ with *U* and *V* being two unitary matrices and Σ being an *n* × *n* diagonal matrix. We generate *U* and *V* with the Matlab code


\begin{eqnarray*} U&=&\tt {orth(randn(m,n)}\\ && \tt +\, \tt {randn(m,n)*1i)}, \end{eqnarray*}

\begin{eqnarray*} V&=&\tt {orth(randn(n,n)}\\ &&\tt +\, \tt {randn(n,n)*1i)}. \end{eqnarray*}

For Σ, we first generate a diagonal matrix using the code


\begin{eqnarray*} \tt {diag} \tt {(sort(rand(n,1),^{\prime }descend^{\prime }))} \end{eqnarray*}

and then rescale this matrix such that its maximum diagonal element is 1; we keep the top *r* = ⌊*n*/8⌋ elements unchanged and further scale down the remaining *n* − *r* elements by a factor of 0.01. This ensures that *A* is of nearly low rank *r*.

We carry out the simulations on a Windows computer with 3.2 GHz CPUs and 32 GB memory. The algorithm accuracy is evaluated by


\begin{eqnarray*} \mathrm{ER}_{\rm SVD}=\frac{\Vert A-\tilde{A}\Vert _{F}}{\Vert A\Vert _{F}}, \\ \mathrm{ER}_{\rm inverse}=\frac{\Vert C(I+A)-I_{m}\Vert _{F}}{\sqrt{m}} \end{eqnarray*}

for the SVD and inverse problems, respectively. Here $\tilde{A}$ denotes the low-rank approximation of *A* based on the truncated SVD and *C* denotes the approximated inverse of (*I* + *A*). In simulations, we observe that Algorithms 3 and 4 consistently lead to ER_SVD_ ≈ 10^−12^ and ER_inverse_ ≈ 10^−12^ over all sizes of *A* under our consideration. Their accuracy matches that obtained by the standard Matlab functions SVD and inverse.

**Figure 1.**
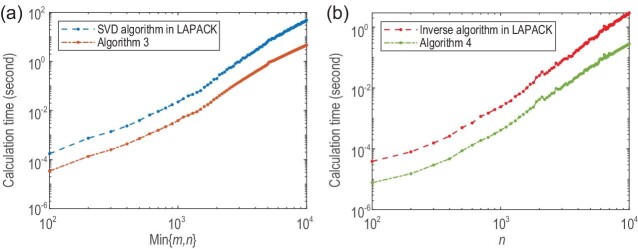
Time (in seconds) to carry out the computational tasks on a nearly low-rank matrix *A* with *m* rows and *n* columns: (a) solving the SVD of *A*; (b) solving the inverse of (*I* + *A*) with *m* = *n*.

In Fig. 1, we report the run times (in seconds) of Algorithms 3 and 4 over varying sizes of *A*. It is seen that the new algorithms are about 5–10 times faster than the algorithms in LAPACK. This justifies the high efficiency of the proposed algorithms.

## FUTURE RESEARCH

In this work, we proposed new algorithms for efficiently solving the SVD and inverse of a nearly low-rank matrix. The proposed algorithms effectively reduce the traditional computational complexity from *O*(*mn*^2^) down to *O*(*mnr*); this leads to a significantly *O*(*n*/*r*) times speedup. The proposed algorithms are accurate and stable; they do not rely on strong structure or sparsity requirements. These properties make the new algorithms attractive in practice.

The techniques in the current work can be extended to other matrix

computation tasks. For example, the ‘online correction’ property of the QB decomposition can be used to solve the linear system of equations in a steaming manner. Algorithm 2 can be geared to efficiently solve the product of two low-rank matrices. Algorithm 4 can be modified to solve high-dimensional ridge regression with a huge number of features.

The current study focuses on the conventional setup, where matrix computation is carried out on a single computer [3–6]. To meet the requirement of the big data era [1], it would be promising to extend the proposed algorithms to the distributed or parallel setup.

## Supplementary Material

nwad083_Supplemental_File
