# Supplementary material for: Fast algorithms for singular value decomposition and the inverse of nearly low-rank matrices
Source: Natl Sci Rev. 2023 Mar 25;10(6):nwad083. doi: 10.1093/nsr/nwad083 (PMC10246826; doi:10.1093/nsr/nwad083)
Supplement: nwad083_Supplemental_File [file nwad083_supplemental_file.pdf]

APPENDIX

We give proofs of Theorems 1 and 2 as follows.

Proof of Theorem 1

Proof. Let $B = \{b_{ij}\}$ be an $r \times r$ matrix with b_{ij} being its (i, j)th element for $i, j \in \{1, \dots, r\}$. Applying QB decomposition to sequence $\{q_1, Aq_1, \dots, Aq_r\}$, we have $b_{ii} = q_i^H Aq_i$ and $b_{ij} = q_i^H Aq_j$. Thus,

$$\begin{aligned}
&q_1 = q_1,\ b_{21}q_2 = Aq_1 - b_{11}q_1,\\
&b_{32}q_3 = Aq_2 - b_{12}q_1 - b_{22}q_2,\\
&\qquad\qquad\vdots\\
&b_{r,r-1}q_n = Aq_{r-1} - b_{1,r-1}q_1 - b_{2,r-1}q_2\\
&- \cdots - b_{r-1,r-1}q_{r-1},\\
&0 = Aq_r - b_{1r}q_1 - b_{2r}q_2 - \cdots - b_{rr}q_r.
\end{aligned}$$

This implies that

$$\begin{aligned}
&[q_1, Aq_1, \dots, Aq_r] = Q[e_1; B]\\
&= [q_1, \dots, q_r] \begin{bmatrix} 1 & b_{11} & \dots & b_{1,r-1} & b_{1r} \\ 0 & b_{21} & \dots & b_{2,r-1} & b_{2r} \\ 0 & 0 & \dots & b_{3,r-1} & b_{3r} \\ \vdots & \vdots & \ddots & \vdots & \vdots \\ 0 & 0 & \dots & b_{r,r-1} & b_{rr} \end{bmatrix}.
\end{aligned}$$

By definition, we have

$$b_{21}q_2 = Aq_1 - b_{11}q_1, \langle b_{21}q_2, q_1 \rangle = b_{11} - b_{11} = 0,$$

where $\langle \cdot \rangle$ denotes the inner product of two vectors. This indicates that $q_2 \perp q_1$.

Similarly, we have

$$\begin{aligned}
&b_{32}q_3 = Aq_2 - b_{22}q_2 - b_{12}q_1,\\
&\langle b_{32}q_3, q_2 \rangle = b_{22} - b_{22} = 0,\\
&\langle b_{32}q_3, q_1 \rangle = \langle q_2, Aq_1 \rangle - b_{12} = 0.
\end{aligned}$$

This indicates that $q_3 \perp q_2$, $q_3 \perp q_1$ and $b_{13} = \langle q_3, Aq_1\rangle = \langle q_3, b_{21}q_2 + b_{11}q_1\rangle = 0$. With the same the technique, one can show that $q_i \perp q_j$ for $i, j \in \{1, \ldots, r\}$ with $i \neq j$ and $b_{ij} = 0$ for any $i + 2 \leq j \leq r$.

Therefore, we have

$$[q_1, Aq_1, \ldots, Aq_r] = [q_1, q_2, ..., q_r]\times$$

$$\begin{pmatrix} 1 & \alpha_1 & \beta_1 & 0 & \cdots & 0 \\ 0 & \beta_1 & \alpha_2 & \beta_2 & \cdots & 0 \\ 0 & 0 & \beta_2 & \alpha_3 & \cdots & 0 \\ \vdots & \vdots & \vdots & \vdots & \vdots & 0 \\ 0 & \cdots & \cdots & \cdots & \alpha_{r-1} & \beta_{r-1} \\ 0 & \cdots & \cdots & \cdots & \beta_{r-1} & \alpha_r \end{pmatrix},$$

where $\alpha_i = b_{ii}(i = 1, \ldots, r)$ and $\beta_j = b_{j+1,j} = b_{j,j+1}(j = 1, \ldots, r-1)$. Since $Q = [q_1, q_2, \ldots, q_r]$ is orthogonal, the above equation implies $A = QBQ^H$, where B is a tridiagonal matrix. The theorem is therefore proved. □

Proof of Theorem 2

Proof. Recall that

$$\alpha_i = q_i^H M q_i \text{ and } \beta_i = q_i^H M q_{i+1}$$

are the diagonal and first sub-diagonal elements of B, as defined in Algorithm 2. Let

$$r_i = (I - \sum_{j=1}^{i} q_j q_j^H) M q_i.$$

We have $\beta_i = \|r_i\|$ and $q_{i+1} = r_i/\beta_i$. It suffices to show that

$$\begin{cases} \beta_i \neq 0 \text{ for } 1 \leq i < n_0, \\ \beta_i = 0 \text{ for } i \geq n_0. \end{cases} \tag{A.1}$$

To this end, we first verify A.1 for $i = 1$. Since q_1 satisfies condition (1), we naturally have

$$\beta_1 = \|r_1\| = \|Mq_1 - \alpha_1 q_1\| \neq 0 \tag{A.2}$$

and $q_2 \neq 0$.

We next show that A.1 holds for $i = 2$. By the definition of Lanczos sequence, we have

$$\beta_1 q_2 = (I - q_1 q_1^H) M q_1 \neq 0,$$

and

$$\begin{aligned}\beta_2 q_3 &= (I - \sum_{j=1}^{2} q_j q_j^H) M q_2 \\ &= (I - q_1 q_1^H) M q_2 - \alpha_2 q_2 \\ &= (I - q_1 q_1^H) M q_2 - \alpha_2 \frac{\beta_1 q_2}{\beta_1} \\ &= (I - q_1 q_1^H)(M q_2 - \frac{\alpha_2}{\beta_1} M q_1).\end{aligned}$$

Now assume that $q_3 = 0$, which implies

$$M q_2 = \beta_1 q_1 + \alpha_2 q_2 \neq 0.$$

Since $q_1 \perp q_2$, we have $M q_2 \neq 0$. Let $M_r^{1/2} = \sum_{j=1}^{r} \lambda_j^{1/2} u_j u_j^H$, where $r = \text{rank}(M)$ and u_j are the eigenvectors of M. It is clear that $M = M_r^{1/2} M_r^{1/2}$. Thus,

$$\begin{aligned}\|M_r^{1/2} q_2\|^2 = \langle q_2, M q_2 \rangle &= \langle M q_2, \frac{\alpha_2}{\beta_1} q_1 \rangle \\ &= \langle M_r^{1/2} q_2, M_r^{1/2} (\frac{\alpha_2}{\beta_1} q_1) \rangle.\end{aligned} \tag{A.3}$$

Since $M q_2 \neq 0$, we also have $M^{1/2} q_2 \neq 0$. Equation (A.3) then implies that

$$M_r^{1/2} q_2 = M_r^{1/2} (\frac{\alpha_2}{\beta_1} q_1)$$

or

$$M_r^{1/2} q_2 = -M_r^{1/2} (\frac{\alpha_2}{\beta_1} q_1).$$

Therefore, we have

$$M(q_2 \pm \frac{\alpha_2}{\beta_1} q_1) = 0,$$

which indicates that

$$q_2 \pm \frac{\alpha_2}{\beta_1} q_1 \in \mathrm{Kernel}(M)$$

with Kernel(M) being the null space of M. Since M is a Hermitian matrix, we have $\mathbf{C}^n = R(M) \oplus \mathrm{Kernel}(M)$ and $R(M) \perp \mathrm{Kernel}(M)$, where $R(M)$ is the range space of M and $\oplus$ denotes direct sum. It follows that

$$< q_2 \pm \frac{\alpha_2}{\beta_1} q_1, x >= 0 \text{ for any } x \in R(M). \tag{A.4}$$

With $x = q_2$, (A.4) immediately implies that $q_2 = 0$, which further implies $\beta_1 = 0$ by definition. This, however, leads to a contradiction with (A.2). Therefore, the assumption $q_3 = 0$ is false; we conclude $q_3 \neq 0$, which implies that $\beta_2 \neq 0$ as $q_2 \neq 0$.

Using the similar arguments, we can show that $\beta_i \neq 0$ and $q_{i+1} \neq 0$ for $i = 1, \ldots, n_0 - 1$. Thus, the 1st part of A.1 is proved.

We complete the proof by showing the 2nd part of (A.1). Suppose that $\beta_{n_0} \neq 0$. Then, by definition, Mq_{n_0} can not be linearly represented by $\{q_1, \ldots, q_{n_0}\}$. Let Θ denotes the size of the orthogonal basis of a subspace in $\mathbf{C}^n$. Since $q_{n_0+1} \in \mathrm{span}\{q_1, \ldots, q_{n_0}, Mq_{n_0}\}$, we have

$$\begin{aligned}
&\Theta(\mathrm{span}\{q_1, q_2, \ldots, q_{n_0+1}\}) \\
&= \Theta(\mathrm{span}\{q_1, Mq_1, \ldots, Mq_{n_0}\}) \\
&= \Theta(\mathrm{span}\{q_1, Mq_1, \ldots, M^{n_0} q_1\}) \\
&= n_0 + 1.
\end{aligned}$$

This implies that M has $n_0 + 1$ degrees of minimal polynomials; this contradicts with the definition of M. We thus conclude that $\beta_{n_0} = 0$.

Since $q_{n_0} \neq 0$, the definition of β_{n_0} indicates that $q_{n_0+1} = 0$. This further implies $\beta_{n_0+1} = 0$. Following the same argu-

ments, we have $\beta_i = 0$ for $i \geq n_0$, which is the 2nd part of (A.1). The theorem is therefore proved. $\square$
